# Draft Genome Sequence of *Lecanicillium* sp. Isolate LEC01, a Fungus Capable of Hydrocarbon Degradation

**DOI:** 10.1128/MRA.01744-18

**Published:** 2019-04-11

**Authors:** Osman Radwan, Thusitha S. Gunasekera, Oscar N. Ruiz

**Affiliations:** a Environmental Microbiology Group, University of Dayton Research Institute, Dayton, Ohio, USA; b Fuels and Energy Branch, Aerospace Systems Directorate, Air Force Research Laboratory, Wright-Patterson AFB, Ohio, USA; University of Maryland School of Medicine

## Abstract

Lecanicillium sp. isolate LEC01 is adapted to grow in the presence of jet fuel, employing genes involved in the degradation of alkanes and aromatic hydrocarbons. The draft genome is estimated at 31,407,988 bp and has 9,737 proteins, 50.0% G+C content, and high similarity to Lecanicillium sp. strain CCF 5233.

## ANNOUNCEMENT

A filamentous fungus was isolated from a hydrocarbon gas turbine fuel sample and identified as Lecanicillium sp. isolate LEC01, with 99% identity to Lecanicillium sp. strain CCF 5233 based on the 18S rRNA sequence. *Lecanicillium* species are entomopathogenic fungi secreting cell wall-degrading enzymes such as chitinases and lipases (1). Although some entomopathogenic fungi can utilize hydrocarbons (2), there has been no report of *Lecanicillium* species that are capable of metabolizing hydrocarbons. Here, we present the draft genome sequence of LEC01, highlighting genes involved in hydrocarbon adaptation and metabolism.

LEC01 was recovered from the fuel via liquid-liquid extraction (3), followed by serial dilution, plating on potato dextrose (PD) agar, and cultivation at 28°C for 3 days. The hyphal tip method (4) was used to obtain the pure isolate from discrete mycelium. Genomic DNA was isolated by the cetyltrimethylammonium bromide (CTAB) method (5) from a 1-week-old culture of LEC01 in PD broth incubated at 28°C with agitation at 200 rpm. The sequencing library was prepared using the PrepX DNA library kit and Apollo 324 next-generation sequencing (NGS) automatic library prep system (WaferGen, Fremont, CA). The ligated and indexed pre-PCR library was enriched by performing 5 cycles of PCR using the NEBNext high-fidelity 2× PCR master mix. The amplified library was purified using the Apollo PCR cleanup script and AMPure XP beads (Beckman Coulter, Brea, CA) prior to quality control (QC) analysis and DNA sequencing. The Illumina HiSeq 1000 platform was used for whole-genome shotgun (WGS) sequencing of TruSeq paired-end libraries from the LEC01 genome. WGS resulted in 54,980,257 paired-end reads with a length of 100 bp and an estimated genome coverage of 160×. Trimmomatic version 0.36 (6) was used to trim low-quality and short reads using the settings “Leading” with a threshold quality of 5, “Trailing” with a threshold quality of 5, “Slidingwindow” with an average quality of 15 across 4 bp, “Avgqual” with an average read quality of 15, and “Minlen” with a minimal length of 50 bp. The trimmed reads were *de novo* assembled using SPAdes version 3.9.1 (7) with the settings “Careful” and “Only-assembler,” resulting in 31,407,988 bp with 50.0% G+C content. The draft genome contains 794 scaffolds larger than 500 bp, with an *L*_50_ value of 27 contigs and an *N*_50_ value of 303,914 bp. The CEGMA-2.5 program (8) identified 239 out of 248 ultraconserved eukaryotic genes, reflecting that the LEC01 draft genome is 96.37% complete. The RepeatMasker (Open-3.0) program (9) was used for masking repetitive sequences (1.28%) with a setting option of fungal species. Genes were predicted using the AUGUSTUS 3.2.1 program (10) with the parameter of the closest fungal species being Verticillium albo-atrum, resulting in 9,737 protein-coding genes.

BLASTP version 2.8.1 (11) searches against the UniProt database, Amorphotheca resinae reference (accession number MADK00000000), and Aspergillus fumigatus reference (accession number AAHF00000000) using an E value of 1e^−5^ identified 77.7%, 77.1%, and 77.5% of the LEC01 proteins, respectively. A local BLASTP search against the Transporter Classification Database (TCDB; http://www.tcdb.org/) resulted in 1,793 different transporters, including those of the major facilitator superfamily (MSF) and ATP-binding cassette (ABC), with possible involvement in the extrusion of toxic compounds. Resistant-nodulation-division (RND) efflux pump, MSF, and ABC transporters may contribute to microbial adaptation to fuel (12–15).

KEGG database and BLASTP searches identified important proteins involved in hydrocarbon degradation and insect infectivity. Chitinase, chitosanase, cutinase, hydrolase, and lipase are examples of proteins in LEC01 that may contribute to insect host infection. The ability of LEC01 to thrive in hydrocarbon fuels is supported by the identification of proteins involved in the biodegradation of *n*-alkanes and aromatics, including cytochrome P450 alkane hydroxylase, cytochrome P450 benzoate 4-monooxygenase, salicylate hydroxylase, dioxygenases, succinate dehydrogenase, and catechol 1,2-dioxygenase. This genome will help in elucidating the mechanisms underlying fungal adaptation to hydrocarbons.

### Data availability.

This whole-genome shotgun project was deposited at DDBJ/ENA/GenBank under the accession number NIWZ00000000. Raw sequences were deposited in the NCBI SRA database under accession number SRP159109.
